# A prospective observational study of on-treatment plasma homocysteine levels as a biomarker of toxicity, depression and vitamin supplementation lead-in time pre pemetrexed, in patients with non-small cell lung cancer and malignant mesothelioma

**DOI:** 10.1371/journal.pone.0225509

**Published:** 2019-11-25

**Authors:** Anna Minchom, Daisy Mak, Ranga Gunapala, David Walder, Rajiv Kumar, Nadia Yousaf, Andrew Hodgkiss, Jaishree Bhosle, Sanjay Popat, Mary E. R. O’Brien

**Affiliations:** 1 Lung Cancer Unit, Royal Marsden NHS Foundation Trust, Sutton, United Kingdom; 2 Lung Cancer Unit, Royal Marsden NHS Foundation Trust, London, United Kingdom; 3 Adult Psychological Support Service, Royal Marsden NHS Foundation Trust, Sutton, United Kingdom; Princess Margaret Cancer Centre, CANADA

## Abstract

**Objectives:**

Vitamin supplementation reduces pemetrexed toxicity. Raised plasma homocysteine reflects deficiency in vitamin B12 and folate, and is suppressed by supplementation. This observational study of 112 patients receiving pemetrexed-based chemotherapy assessed homocysteine levels after 3 weeks of vitamin supplementation, hypothesising high levels would correlate with ongoing deficiency, thus increased toxicity.

**Material and methods:**

Primary endpoint was the composite of proportion of patients with treatment delay/ dose reduction/ drug change or hospitalisation during the first six weeks of chemotherapy, comparing those with normal plasma homocysteine (successfully supplemented, SS) and those with high homocysteine (unsuccessfully supplemented, USS). Secondary endpoints included toxicity and analyses for depression. Post-hoc analysis examined correlation between interval of vitamin and folate supplementation and pemetrexed on primary endpoint and grade 3–4 toxicities.

**Results:**

Eighty-four patients (84%) were successfully supplemented (SS group). The proportion of patients undergoing a treatment delay/ dose reduction/ drug change or hospitalisation in SS group was 44.0% (95% confidence interval [CI] 33.2%–55.3%) and in USS group was 18.8% (95% CI 4.0%–45.6%) (*p* = 0.09). Twelve percent of patients gave a past history of depression however 66% of patients had an on study Hospital Anxiety and Depression (HAD) score of >7. Supplementation status was not associated with depression. The median overall survival (OS) was 11.8 months (95% CI 8.6–16.5) in the SS group and 8.8 months (95% CI 6.6–16.2) in the US group (*p* = 0.5). The number of days (<7 or ≥ 7 days) between vitamin B12 and folate initiation and pemetrexed administration, had no effect on the primary endpoint and grade 3–4 toxicities.

**Conclusion:**

On-treatment homocysteine levels were not a biomarker of toxicity or depression. Standard vitamin supplementation is adequate in the majority of patients receiving pemetrexed. High HAD score were noted in this population giving an opportunity for mental health intervention. The lead-in time for vitamin supplementation can be short.

## Introduction

Globally 1.8 million new lung cancer diagnoses occurred in 2012. It is the leading cause of cancer death worldwide in developed countries, and prognosis for advanced disease remains poor. Chemotherapy is important to control symptoms and prolong survival in patients without a targetable mutation or with resistance to targeted therapies, thus predictors of toxicity and prognosis are useful.

Pemetrexed is a multitargeted antifolate agent, and is a current standard in treating advanced adeno-NSCLC and malignant mesothelioma. Pemetrexed causes grade 3–4 neutropenia and dose delay in a significant proportion of patients[1]. Vitamin B12 and folic acid supplementation significantly reduces drug-related deaths and toxicities[2], and is recommended before and during pemetrexed-containing regimens.

Plasma homocysteine levels correlate with folic acid and vitamin B12 deficiency and folic acid supplementation reduces plasma homocysteine [3]. Studies found patients treated with pemetrexed who had elevated homocysteine level at baseline suffered from severe haematological and gastrointestinal toxicities [4], and those successfully supplemented with vitamins had significantly less neutropenic toxicity with platinum chemotherapy compared to unsupplemented [5]. The normal (interquartile) range for homocysteine, established in cardiovascular population is 7.5–11.5 μ M/L [6–8] A previous study in the oncology population, using these reference ranges, established the risk of pemetrexed toxicity increased in those with homocysteine levels above 11.5 μM/L with a 300% increase in the risk of developing a severe toxicity. In levels below 7.5 μM/L there was a 30% reduction in the risk of developing a severe toxicity[9].

The Hospital Anxiety and Depression Score (HADS) is a validated questionnaire used for screening for anxiety and depression. Depression, being observed in a number of lung cancer patients, could have a biological basis and relate to treatment. In particular, homocysteine, being an *N*-methyl-D-aspartate receptor agonist, causes decreased neurogenesis in the dentate gyrus of the hippocampus [10]. However, a correlation between homocysteine levels and depression has not been established.

This prospective, observational study of patients receiving vitamin B12 and folic acid supplementation with pemetrexed-based chemotherapy aimed to assess homocysteine levels after 21 days of supplementation. We hypothesised that those with a high level of homocysteine would have more toxicity. Thus the 3-week homocysteine level would serve as a predictor of increased chemotherapy-induced toxicity. We also aimed to provide exploratory data on the correlation of homocysteine levels with depression, and investigate whether the interval between vitamin B12 and folate supplementation and pemetrexed administration affects toxicities.

## Materials and methods

### Trial design

This observational single arm study recruited patients with advanced NSCLC and mesothelioma suitable for palliative, pemetrexed-based chemotherapy. Patient recruitment spanned from 25^th^ February 2014 to 25^th^ September 2015. Follow-up was carried out until one year after the entry of the last patient (i.e. 25^th^ September 2016). The study was approved by the Wales Research Ethics Committee 4 under the approval number 13/WA/0356. All patients gave full written consent.

Oral folic acid 400 mcg daily and vitamin B12 1000 mcg intramuscular were commenced at least 1 week before the 1^st^ cycle; vitamin B12 was repeated every 9 weeks during treatment, as per local guidelines.

A homocysteine sample was taken on cycle 2 day 1 of chemotherapy, i.e. after at least 3 weeks of supplementation. The HADS questionnaire was introduced following a protocol amendment after 56 patients had been recruited.

### Eligibility

Inclusion criteria included age greater than 18 years, advanced stage disease (UICC version 7 stage IIIB/IV), receiving combination pemetrexed-based treatment (with cisplatin 50–75 mg/m^2^ or carboplatin AUC 5/6) or single agent pemetrexed. Patients had to be able to take vitamin B12 and folic acid supplementation and have an estimated life expectancy of at least 12 weeks.

Exclusion criteria included inability to take vitamin supplementation and patients receiving concomitant radiotherapy. Patients could have received previous lines of chemotherapy as long as no vitamin supplementation was given.

Patients were recruited in the Royal Marsden NHS Foundation Trust outpatient clinics.

### Outcomes

The primary endpoint was to identify difference in proportion of patients undergoing a treatment delay/ dose reduction/ drug change or hospitalisation during the first six weeks of chemotherapy. Patients who after at least 21 days of vitamin B12 and folic acid supplementation show normal plasma levels of homocysteine (defined as <10 micromol/L; successfully supplemented, SS group) were compared to patients that showed abnormal plasma levels (defined as ≥10 micromol/L; unsuccessfully supplemented, USS group).

Secondary endpoints include identifying differences in number of patients with grade 3–4 toxicities (except alopecia; CTCAE version 4.0) and OS between the SS and USS groups. There were exploratory analyses for depression, and post-hoc analyses to examine whether the interval between vitamin B12 and folate supplementation and pemetrexed administration will affect the primary endpoint and grade 3–4 toxicities.

### Sample size and interim analysis

We predicted 50% of the study population would be in the USS group, and 40% of USS group and 15% of the SS group would show a treatment delay/ dose reduction/ drug change or hospitalisation rate. To detect a difference between the two groups with 80% power and 2-sided alpha of 5%, 98 patients were required.

For the secondary endpoints we predicted that 30% of the USS group and 15% of the SS group would show a grade 3/4 toxicity rate. To detect a difference between the two groups with 80% power and 2-sided alpha of 5%, a total of 242 patients were required.

It was planned that after the first 98 patients had finished 6 weeks of treatment an interim analysis of the primary end-point would be performed. If the reported *p*-value was significant, recruitment would continue to 242 patients, if not the trial would be closed early and the secondary endpoints reported.

### Statistical methods

All tests were two-sided assessed at 5% significance level. Differences were compared using a Chi-square or Fisher’s exact test. Statistical analysis was performed using SPSS version 24.

## Results

### Recruitment

**Fig 1:**
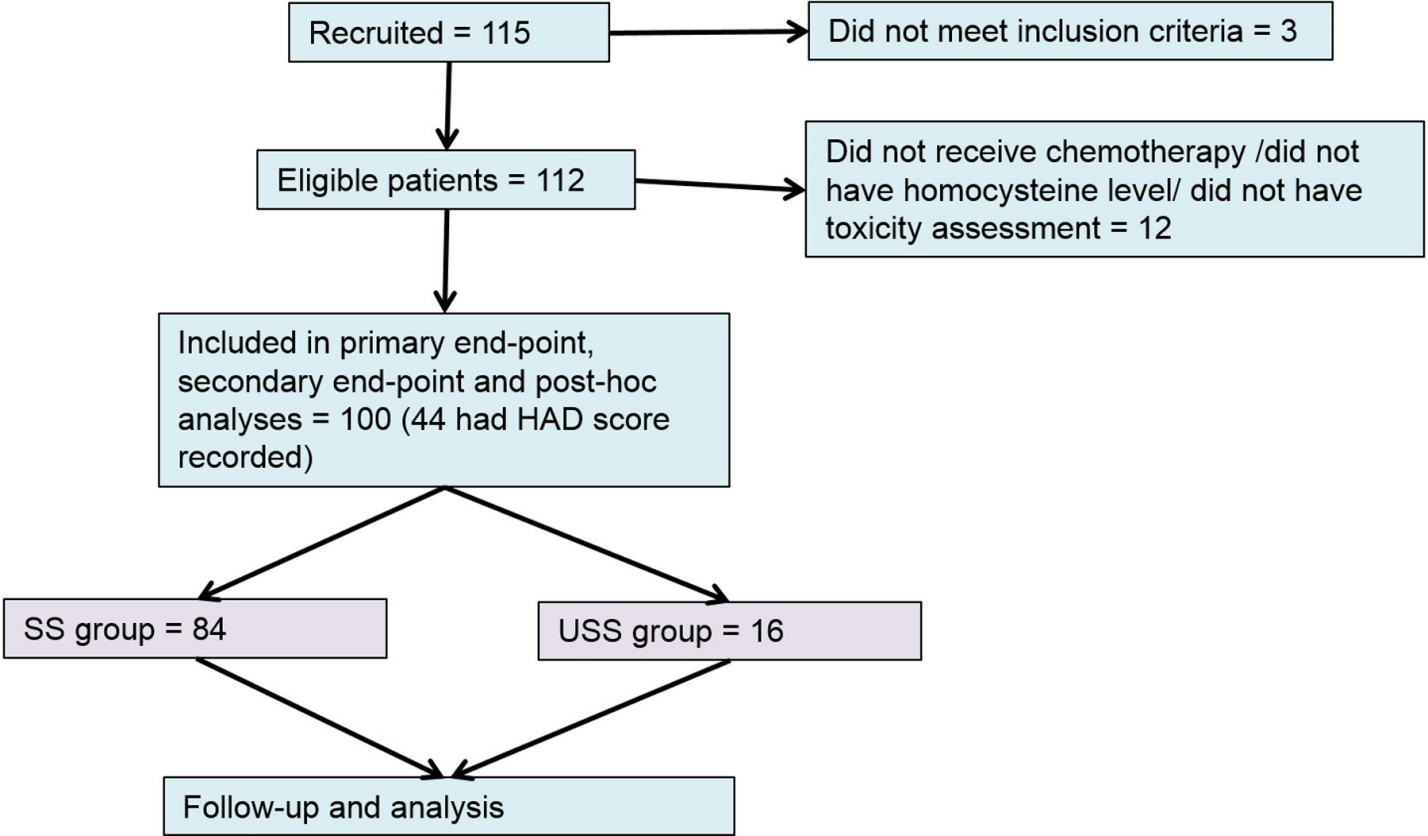
Consort diagram of recruitment, eligibility and end-point analysis.

At the time of the interim analysis 115 patients had been recruited to the trial. Three patients were ineligible having received AUC4 carboplatin. 100 patients who received at least one chemotherapy cycle and had a homocysteine level documented were included in further analysis (Fig 1).

### Baseline data

Fifty-eight percent of patients were female, with a median age of 66 years (range 41–87 years). The performance status (PS) was ECOG 0 in 22.3%, ECOG 1 in 67.9%, and ECOG 2 in 9.8%. A history of depression was reported in 11.7% of patients. There were 17.9% with malignant pleural mesothelioma, the remainder had adeno-NSCLC. The baseline characteristics were well-balanced between USS and SS groups apart from sex (45% men in SS group and 63% men in USS group) and distribution of PS (PS 0:1:2 of 21.3: 67.9: 10.7 in SS group and 25.0: 75.0: 0 in USS group). Only two patients (2%) received single agent pemetrexed, 35% receiving cisplatin-pemetrexed combination and 63% receiving carboplatin-pemetrexed combination.

### Primary endpoint

Among the 100 patients, 84% patients had successful supplementation with a normal homocysteine level (SS group).

In the SS group the proportion of patients undergoing a treatment delay/ dose reduction/ drug change or hospitalisation was 44.0% (37/84 patients; 95% confidence interval [CI] 33.2%–55.3%), while that in the USS group was 18.8% (3/16 patients; 95% CI 4.0%–45.6%) (*p* = 0.09) (Table 1). The trial was terminated at the interim analysis and secondary end-points reported.

**Table 1:** Primary end-point of treatment delay/ dose reduction/ drug change or hospitalisation in successfully supplemented (SS) group and unsuccessfully supplemented (USS) group.

	SS group	USS group	Fisher’s exact test
**Primary End Point:** **Treatment delay/** **dose reduction/** **drug change** **or hospitalisation**	44.0% (37/84 patients; 95% CI 33.2% - 55.3%)	18.8% (3/16 patients; 95% CI 4.0% - 45.6%)	*p* = 0.09
**Treatment delay**	21.4% (18/84 patients; 95% CI 13.2% - 31.7%)	12.5% (2/16 patients; 95% CI 1.6% - 38.3%)	*p* = 0.52
**Dose reduction**	20.2% (17/84 patients; 95% CI 12.2% - 30.4%)	12.5% (2/16 patients; 95% CI 1.6%– 38.3%)	*p* = 0.73
**Drug change**	13.1% (11/84 patients; 95% CI 22.2% - 67.2)	18.8% (3/16 patients; 95% CI 4.0% - 45.6%)	*p* = 0.69
**Hospitalisation**	16.7% (14/84 patients; 95% CI 26.4% - 94.2%)	18.8% (3/16 patients; 95% CI 4.0% - 45.6%)	*p* = 1.00

### Secondary endpoints

There was no statistically significant difference in grade 3–4 toxicities between patients with a PS of ECOG 0/1 vs. those with ECOG 2 (16% of ECOG 0/1 patients vs 11% of ECOG 2 patients had grade 3–4 toxicities, *p* = 0.999). In the SS group, the proportion of patients with a grade 3–4 toxicity was 14.5% (12/83 patients; 95% CI 7.7%–23.9%), while that in the USS group was 18.8% (3/16 patients; 95% CI 4.0%–45.6%) (*p* = 0.70).

OS was measured from cycle 1 day 1 of chemotherapy. Median OS of SS group was 11.8 months (95% CI 8.6–16.5 months) and 8.8 months in the USS group (95% CI 6.6–16.2 months) (*p* = 0.5; Log Rank test).

In the population with HAD score recorded (44 patients), 66% (29/44 patients) had HAD score >7. In SS group, the proportion of patients with a HAD score >7 was 63.9% (23/36 patients; 95% CI 46.2%–79.2%) vs 75% (6/8 patients; 95% CI 34.9%–96.8%) in the USS group (*p* = 0.70).

### Post-hoc analyses

The median interval between vitamin B12 and folate supplementation and pemetrexed administration was 7 days (range, 0–25 days). The duration of vitamin B12 and folate before pemetrexed, defined as <7 days (53 patients) and ≥7 days (47 patients), had no correlation with SS and USS grouping (91% vs 77% with interval of <7 and ≥7 days had successful supplementation, *p* = 0.10). We further found that the number of days (<7 or ≥ 7 days) between vitamin B12 and folate and pemetrexed administration had no effect on the primary endpoint as well as G3-4 toxicities (Table 2). We repeated the analysis, redefining the interval as ≤1 and >1 day, and still found that no significant association between the interval with the primary endpoint and grades 3–4 toxicities.

**Table 2:** Primary end-point of treatment delay/ dose reduction/ drug change or hospitalisation in patients receiving vitamin B12 and folate <7 days and ≥7 days.

	No. of days between vitamin B12/folate prior to pemetrexed		Fisher’s exact test
	<7 days N = 53	≥7 days N = 47	
**Primary End Point:** **Treatment delay/** **dose reduction/** **drug change** **or hospitalisation**	39.6% (95% CI, 26.5–54.0%)	40.4% (95% CI, 26.4–55.7%)	*p* = 0.94
**Treatment delay**	22.6% (95% CI, 12.3–36.2)	17.0% (95% CI, 7.6–30.8%)	*p* = 0.48
**Dose reduction**	18.9% (95% CI, 9.4–32.0%)	19.2% (95% CI, 9.1–33.3%)	*p* = 0.97
**Drug change**	9.4% (95% CI, 3.1–20.7%)	19.2% (95% CI, 9.1–33.3%)	*p* = 0.25
**Hospitalisation**	15.1% (95% CI, 6.7–27.6%)	19.2% (95%, CI 9.1–33.3%)	*p* = 0.59
**G3/4 toxicities**	15.4% (95% CI, 6.9–28.1)	14.9% (95% CI, 6.2–28.3%)	*p* = 0.95

## Discussion

In this study appropriate levels of supplementation, as defined by a normal homocysteine level after 3 weeks of Vitamin B12 and folic acid, were seen in the majority (84%) of patients. We conclude that standard vitamin supplementation is adequate in the majority of patients receiving pemetrexed. This high rate of appropriate supplementation confounded results as the USS group contained a small number of patients. With that caveat, high levels of homocysteine did not correlate with increased toxicity and therefore in this patient group is unlikely to be a useful biomarker to identify patients at an increased risk of toxicity. We did not include baseline homocysteine measurement in this study given this correlation has been previously well explored in studies [5, 9], our aim being to establish if on-treat homocysteine is a viable marker of toxicity.

While 15% of the study population experienced grade 3/4 toxicity, 40% underwent a delay in their chemotherapy, change in chemotherapy dose or regimen or hospitalisation during the first 6 weeks of their chemotherapy. This composite end-point reflects those experiencing a toxicity that impaired their ability to continue with the planned schedule or resulted in hospitalisation. We would propose that the measurement of grade of toxicity alone may not adequately reflect how patients are coping with chemotherapy.

There was no difference in the rates of grade 3/4 toxicities between patients with PS of ECOG 0/1 patients and those with ECOG 2. Though original trials of pemetrexed in lung cancer excluded those of PS 2 [5], a subsequent trial demonstrated that those of PS 2 derived an OS, progression-free survival and response rate benefit from pemetrexed (versus pemetrexed-carboplatin) with tolerable toxicity [11]. Our data supports the tolerability of pemetrexed in PS 2 patients based on grade 3/4 toxicity, though the definition of tolerability from grade 3/4 toxicity alone may be somewhat limited, as discussed. The study was not powered for OS.

At present, the standard supplementation regimen prior to pemetrexed includes vitamin B12 1,000 mcg given intramuscularly and oral folic acid 400 mcg, administered at least 5–7 days prior to the first dose of pemetrexed. In the current study, we found that the interval between supplementation of vitamin B12 and folate and first dose of pemetrexed (<7 vs ≥ 7 days, and ≤1 vs >1 day) did not significantly alter the primary end-point and grade 3–4 toxicities. Multiple prospective and retrospective studies [12, 13, 14] have addressed the issue of lead-in time of vitamin supplementation before pemetrexed, and have also found that giving vitamin supplementation closer to the day of starting pemetrexed, had no significant effect on grades 3–4 cytopenias, and non-haematological toxicities. Our results suggest that the lead-in time for vitamin supplementation could be shortened to 1 day before first cycle of pemetrexed. This is especially relevant for convenient scheduling of chemotherapy and avoiding delays when disease is rapidly progressive.

In our cohort, only 11.7% of patients reported depression at baseline. The HAD score was introduced following a protocol amendment midway through the trial but, of those who had a HAD recorded (n = 44), 66% had a HAD score > 7, indicative of depression. Thus the HAD score appears to substantially increases the pick-up of depression in this patient group, potentially allowing earlier intervention. Within the small numbers, no relationship on trend of HAD score to homocysteine level could be identified.

In conclusion, on-treatment homocysteine levels were not shown to be a biomarker of toxicity in this study. Standard vitamin supplementation is probably adequate in the majority of patients. Despite vitamin supplementation there continues to be issues with the deliverability of pemetrexed-based chemotherapy in this patient group. The lead-in time of vitamin supplementation can be shortened. The HAD score suggest a high rate of subclinical or undiagnosed depression, affording an opportunity for potential mental health intervention.

## Supporting information

S1 Study Protocol (DOC)
