# Supplementary material for: A prospective observational study of on-treatment plasma homocysteine levels as a biomarker of toxicity, depression and vitamin supplementation lead-in time pre pemetrexed, in patients with non-small cell lung cancer and malignant mesothelioma
Source: PLoS One. 2019 Nov 25;14(11):e0225509. doi: 10.1371/journal.pone.0225509 (PMC6877184; doi:10.1371/journal.pone.0225509)
Supplement: S1 Study Protocol — (DOC) [file pone.0225509.s001.doc]

**PROSPECTIVE EVALUATION OF THE ROLE OF HOMOCYSTEINE LEVELS IN BLOOD AS A PREDICTIVE BIOMARKER OF TOXICITY AND PROGNOSTIC BIOMARKER IN LUNG CANCER AND MESOTHELIOMA**

**PATIENTS RECEIVING VITAMIN B12 AND FOLATE SUPPLEMENTATION WITH THEIR CHEMOTHERAPY – an observational study**

**SHORT TITLE: HC 21**

**Chief Investigator**

Mary O’Brien

Statistician

Ranga Gunapala

Royal Marsden Hospital,

Downs Road, Sutton SM2 5PT,

Fulham Road, London SW3 6JJ

Tel: 020 8661 3278

Mary.obrien@rmh.nhs.uk

Version 3, 17^th^ October 2014

REC No :

Sponsor Ref: CCR4034

**PROTOCOL SYNOPSIS**

| TITLE | PROSPECTIVE EVALUATION OF THE ROLE OF HOMOCYSTEINE LEVELS IN BLOOD AS PREDICTIVE BIOMARKER OF TOXICITY AND AS A PROGNOSTIC BIOMARKER IN LUNG CANCER AND MESOTHELIOMA RECEIVING VITAMIN B12 AND FOLATE SUPPLEMENTATION WITH THEIR CHEMOTHERAPY- an observational study |
| --- | --- |
| STUDY DESIGN | This will be an observational single arm study with 242 patients included. Patients affected by NSCLC or mesothelioma (MPM) suitable for palliative pemetrexed-based chemotherapy with vitamin B12 and folate supplementation will have a plasma homocysteine level measure at day 21 only. This result will be correlated with toxicity and outcome. Exploratory data regarding rates of depression with also be collected, |
| STUDY OBJECTIVES |  |
| - Primary | To identify differences in proportion of patients undergoing a treatment delay/ dose reduction/ drug change or hospitalisation during the first 6 weeks of chemotherapy, between patients who after at least 21 days of vitamin B12 and folate supplementation show normal plasma levels of homocysteine (<10 micromol/L) compared to patients that still show abnormal plasma levels (≥10 micromol/L). Treatment delay for administration reasons will be ignored |
| - Secondary | 1. To identify differences in number of patients with grade 3-4 toxicities (except alopecia) or death during the first 6 weeks of chemotherapy, between patients who after at least 21 days of vitamin B12 and folate supplementation show normal plasma levels of homocysteine (<10 micromol/L) and patients that still show abnormal plasma levels (≥ 10 micromol/L).  2. To describe the total number of grade 3-4 toxicities experienced during the first 6 weeks of chemotherapy in those with homocysteine level <10 micromol/l and those with homocysteine level ≥10 micromol/l.  3. To investigate whether homocysteine plasma levels measured (<10 micromol/l versus 10 micromol/l or greater) after adequate vitamin B12 and folate supplementation could predict outcome by comparing median Overall Survival (OS).  4. Toxicity and survival according to performance status 0, 1 versus 2  5. Survival description of NSCLC compared to mesothelioma  6. Survival description of whole group consented to include all patients even those with/without homocysteine level but no or less than 6 weeks toxicity data.  7. To compare depression levels (rated as HAD-D >7) in those with homocysteine level <10 micromol/l and those with homocysteine level 10micromol/l or greater  8. To compare total length of stay in hospital (summated for all hospital stays over the six week period) in those with homocysteine level < 10 micromol/l and those with homocysteine levels >/= 10 micromol/l. |
| STUDY POPULATION CRITERIA |  |
| - Inclusion Criteria | Patients must fulfil ALL the inclusion criteria.   1. Written informed consent 2. Aged ≥ 18 years 3. Histologically or cytologically confirmed non-squamous NSCLC or MPM 4. Stage IIIB/IV disease. 5. ECOG PS: 0 – 2. 6. Patients must be suitable to receive pemetrexed-based treatment with Cisplatin 50-75 mg/m^2^ (or Carboplatin AUC 5 or 6) or single agent pemetrexed therapy. 7. Patients must be able to take acid folic and vitamin B12 supplementation as normally required in clinical practice. 8. Estimated life expectancy of at least 12 weeks. |
| - Exclusion Criteria | Patients could not be enrolled in the study if they meet ANY of subsequent exclusion criteria:   1. Pregnant or lactating women 2. Active infection 3. Inability or unwillingness to take vitamin supplementation 4. Serious systemic disorders incompatible with the study at the discretion of the investigator. 5. Patients receiving concomitant radical chemotherapy and radiotherapy (palliative or antalgic local radiotherapy is permitted) 6. Patients may have received previous lines of chemotherapy or targeted therapy as long as no vitamin supplementation was given. |
| PRIMARY ENDPOINT | Proportion of patients undergoing a treatment delay/ dose reduction/ drug change or hospitalisation during the first 6 weeks of chemotherapy |
| SECONDARY ENDPOINT | 1. Proportion of patients with grade 3-4 toxicities (except alopecia), or deathduring the first 6 weeks of chemotherapy.  2. Total number of grade 3-4 toxicities experienced by patients during the first 6 weeks of chemotherapy in those with homocysteine level <10 micromol/l and those with homocysteine level ≥10 micromol/l.  3. To compare overall survival in those with homocysteine levels <10 micromol/l and those with homocysteine level 10 mincormol/l or greater.  4. Toxicity and overall survival according to performance status 0,1 versus 2  5. Overall survival of NSCLC compared to mesothelioma  6. Overall survival of whole group consented to include all patients even those with/without homocysteine level but no or less than 6 weeks toxicity data. This includes all patients registered onto the study including dropouts, who may have died before the blood test at 3 weeks or died before toxicity assessment at 6 weeks.  7. To compare depression levels (rated as HAD-D >7) in those with homocysteine level <10 micromol/l and those with homocysteine level 10micromol/l or greater.  8. To compare total length of stay in hospital (number of days summated for all hospital stays over the six week period) in those with homocysteine level < 10 micromol/l and those with homocysteine levels >/= 10 micromol/l. |

**CONTENTS**

Protocol signature page 8

1. Background 9

2. Rationale 12

3. Study objectives 12

*3.1 Primary objective* 12

*3.2 Secondary objective* 12

4. Study design 13

5. Study population 14

*5.1 Inclusion Criteria*  14

*5.2 Exclusion criteria* 15

6. Study Treatment and Procedures 15

*6.1 Treatment Administration*  15

*6.2 Concomitant Medications*  16

*6.3 Chemotherapy Treatment*  16

*6.4 Treatment of Depression*  16

7. Study Organisation 16

8. Study Procedures 19

9. Study Assessment 20

*9.1 Baseline/Screening assessment* 20

*9.2 Homocysteine assessment* 20

*9.3 Toxicity assessment*  20

*9.4 Depression assessment*  21

*9.5 Post-treatment assessment* 21

10. Evaluation of Outcome 21

*10.1 Primary Endopoint* 21

*10.2 Secondary Endopoints* 21

11. Adverse events 22

*11.1 Adverse event* 22

*11.2 Serious adverse event* 23

*11.3 Suspected unexpected serious adverse reactions (SUSARs)* 23

*11.4 What should be reported?*  24

12. Statistical Considerations 24

*12.1 Sample size estimation* 24

*12.2 Analysis of primary endpoint* 25

*12.3 Analysis of secondary endpoints* 26

*12.4 Timing of analysis*  27

13. References 28

14. Appendices 30

**PROTOCOL SIGNATURE PAGE**

STUDY TITLE: Prospective evaluation of the role of homocysteine levels in blood as predictive biomarker of toxicity and as a prognostic biomarker in lung cancer and mesothelioma receiving vitamin B12 and folate supplementation with their chemotherapy – an observational study (HC 21).

Protocol version 3.0

17^th^ September 2014

Approved by Chief Investigator:

_________________________________________________________

(Dr Mary O’Brien MD FRCP)

Date:

By signing above, the Investigator agrees to adhere to the protocol as outlined and that this study will be conducted in accordance with the Principles of Good Clinical Practice (GCP), the EU and GCP Directives (2001/20/EC; 2005/28/EC) and the Medicines for Human Use (Clinical Trials) and Blood Safety and Quality (Amendment) Regulations 2008 (Statutory Instrument 2008 No. 941)

**1. Background**

**Lung Cancer**

Lung cancer still remains the leading cause of cancer related death and one of the most frequent cancer diagnoses worldwide in both males and females. In the vast majority of cases, lung cancer is staged as locally advanced or metastatic disease and prognosis of these patients is poor. Non-Small Cell Lung Cancer (NSCLC), which includes adenocarcinoma, squamous and large cell carcinoma, accounts for about 85% of lung cancer diagnoses. The identification of specific genetic alteration, such as activating EGFR mutations, ALK rearrangement and, recently, ROS1 rearrangement, together with the availability of specific targeted therapies has dramatically changed prognosis and life expectancy for these patients affected by mutated NSCLC cancer. Unfortunately, NSCLC harbouring targetable genetic alterations are uncommon and, in patients with non-mutated cancer, to date, standard treatment for stage IIIB-IV NSCLC is still based on platinum associated with pemetrexed, in the non-squamous histotype, and platinum combined with vinorelbine, gemictabine, docetaxel or paclitaxel in the squamous subtype. In this setting chemotherapy maintains a palliative role, aiming to control symptoms and prolong survival without deterioration in quality of life. For this reason, in these patients it becomes extremely important to identify reliable predictors of toxicity and prognostic markers.

**Pemetrexed Toxicity and Homocysteine Levels**

Pemetrexed is a multitargeted antifolate agent, able to inhibit dihydrofolate reductase (DHFR), thymidylate synthase (TS) and glycinamide ribonucleotide formyltransferase (GARFT), enzymes involved in DNA synthesis [1]. In the last decade, pemetrexed demonstrated its activity in thoracic malignancies including Malignant Pleural Mesothelioma (MPM). During the clinical development of pemetrexed, drug-related toxicity, with bone marrow suppression, was document in 50% of patients as grade 3 or 4 neutropenia. Other grade 3-4 toxicities reported were febrile neutropenia, diarrhoea and mucositis, which became in some cases life-threatening [2]. The first randomised controlled trials of pemetrexed in combination with cisplatin found 37.5% of patients had a dose delay at some point over their treatment course (a median of 5 cycles give) and 1.5% of patients had dose reduction. [3] A later study of pemetrexed versus pemetrexed and carboplatin in combination had similar results with dose delays occurred in 44.7% of patients on combination pemetrexed and carboplatin and in 20.6% of patients on pemetrexed alone and dose reductions in 3.9% of patient on combination therapy and 2.9% of patients on pemetrexed alone. [4] A close connection was demonstrated between toxicity related to antifolate drugs and folic acid supplementation [5-8].

Given the fact that raised homocysteine was demonstrated to be a useful tool to assess folate deficiency [9,10], a study was conducted to find out a possible correlation between toxicity in patients treated with pemetrexed and pre-treatment plasma level of several markers of folic acid metabolism, including homocysteine. High homocysteine level at baseline correlated significantly with severe haematological and gastrointestinal toxicity and was demonstrated to be a predictive marker of toxicity in patients with MPM and NSCLC treated with pemetrexed [11]. Since folic acid integration demonstrated a lowering effect on plasma homocysteine levels [12], vitamin B12 and folate supplementation was introduced in trials evaluating pemetrexed and this resulted in a significant reduction of drug related deaths and toxicities without affecting drug efficacy [13]. With this evidence, folic acid and vitamin B12 integration has become part of the pemetrexed regimen.

Moreover, in a double-blind randomized trial evaluating chemotherapy with pemetrexed versus placebo, a benefit was demonstrated also in patients not receiving pemetrexed but who also received vitamin B12 and folate supplementation [13] suggesting that all lung cancer patients may have a folate deficiency that if corrected would result in less toxicity with platinum containing regimens. A study was conducted, at the Royal Marsden, in order to assess the impact of addition of vitamin supplementation on toxicity in patients treated with platinum (without pemetrexed) and to determine the potential role of homocysteine level in predicting toxicity. Patients with all subtypes of lung cancer (including small cell lung cancer) and mesothelioma were enrolled. Baseline homocysteine levels did correlate with toxicity in the chemotherapy alone group with a significant higher neutropenic toxicity rate when compared to vitamin B12 and folate supplemented patients (p=0.02). An advantage in median overall survival in patients with homocysteine levels at 2 weeks after supplementation < 10 micromol/L (successful supplementation, SS) was noted when compared with patients with homocysteine levels ≥ 10 micromol/L (unsuccessfull supplementation, UNSS) (15.7 vs 9.2 mths). However, this trial was not powered for survival differences, but it suggests that patients with lung cancer and mesothelioma, regardless of receiving pemetrexed or non-pemetrexed regimens, may benefit from this simple and low cost vitamin supplementation.

Cisplatin and pemetrexed is currently our most commonly used regimen in the non-squamous NSCLC practice. Vitamin B12 and folic acid are routinely given in patients receiving pemetrexed. This is also the regimen used in MPM.

Homocysteine Levels and Depression

Temel et al showed that 38% of NSCLC patients having standard care were depressed 3 months after diagnosis (score >7 on HADS-D subscale). [14] This depression rate was significantly reduced, from 38% to 16%, if early, high quality palliative care was added. Others have found major depressive illness occurring a rate of 13.1% in lung cancer patients. [15] There are several biological mechanisms by which advanced NSCLC and its treatment can provoke psychopathology. The decreased availability of SAM (S-adenosylmethionine) and biopterin leads to decreased activation of tryptophan and tyrosine hydroxylase, and less monoamine neurotransmitter synthesis. In addition, homocysteine is an NMDA receptor agonist, an excitotoxin causing decreased neurogenesis in the granule cells of the dentate gyrus of the hippocampus. The resulting depressed mood may be ameliorated by fluoxetine. [16]

The Hospital Anxiety and Depression Score (HAD score) is a 14 point questionnaire used as a screening tool for anxiety and depression. First devised in 1983 it has been extensively validated since then. [17-18}

**2. Rationale**

We propose an observational study to look at all patients receiving vitamin B12 and folate supplementation along with their chemotherapy to assess the homocysteine level after 3 weeks (21 days) of supplementation i.e. prior to cycle no 2. We hypothesize that those with a high level of homocysteine at this point (UNSS) will have more toxicity and shorter survival than those with a low or </=10 level. If the prognostic and predictive value of homocysteine is confirmed then further trials on high dose vitamin supplementation can be proposed.

The HAD score will be administered at cycle 2, day 1. A measure of the feasibility of the administration of the HAD will inform further trial design in this patient cohort of an interventional trial of early psychiatric input and provide exploratory data on the correlation of homocysteine levels with depression.

This study will be conducted in compliance with GCP guidelines.

**3. Study objectives**

3.1 Primary objective:

To identify differences in proportion of patients undergoing a treatment delay/ dose reduction/ drug change or hospitalisation during the first 6 weeks of chemotherapy, between patients who after at least 21 days of vitamin B12 and folate supplementation show normal plasma levels of homocysteine (<10 micromol/L) compared to patients that still show abnormal plasma levels (≥10 micromol/L). Treatment delay for administration reasons will be ignored

3.2. Secondary objectives:

1. To identify differences in number of patients with grade 3-4 toxicities (except alopecia) or death during the first 6 weeks of chemotherapy, between patients who after at least 21 days of vitamin B12 and folate supplementation show normal plasma levels of homocysteine (<10 micromol/L) and patients that still show abnormal plasma levels (≥ 10 micromol/L).

2. To describe the total number of grade 3-4 toxicities experienced during the first 6 weeks of chemotherapy in those with homocysteine level <10 micromol/l and those with homocysteine level ≥10 micromol/l.

3. To investigate whether homocysteine plasma levels measured (<10 micromol/l versus 10 micromol/l or greater) after adequate vitamin B12 and folate supplementation could predict outcome by comparing median Overall Survival (OS).

4. Toxicity and survival according to performance status 0, 1 versus 2

5. Survival description of NSCLC compared to mesothelioma

6. Survival description of whole group consented to include all patients even those with/without homocysteine level but no or less than 6 weeks toxicity data.

7. To compare depression levels (rated as HAD-D >7) in those with homocysteine level <10 micromol/l and those with homocysteine level 10micromol/l or greater

8. To compare total length of stay in hospital (summated for all hospital stays over the six week period) in those with homocysteine level < 10 micromol/l and those with homocysteine levels >/= 10 micromol/l.

**4. Study Design**

This will be an observational single arm study with 242 patients included. Patients with advanced NSCLC and mesothelioma (MPM) suitable for palliative pemetrexed-based chemotherapy with vitamin B12 and folate supplementation for at least 21 days will be enrolled at any point from day 1 to day 21.

No extra clinical investigations will be needed. All procedures will be performed as per Table 1.

Table1. Schedule of Events

|  | **Screen/baseline** | **Cycle 1** | **Cycle 2** | **Cycle 3** |
| --- | --- | --- | --- | --- |
|  |  | **day 1** | **day 1** | **day 1** |
| **DATE** |  |  |  |  |
| Assess inclusion/exclusion criteria | X | X |  |  |
| Written informed consent | X | X |  |  |
| Document toxicities | X |  | X | X |
| Homocysteine level |  |  | X |  |
| HAD-D score |  |  | X |  |

**5. Study Population**

Patients affected by NSCLC or mesothelioma (MPM) suitable for palliative pemetrexed-based chemotherapy will be enrolled in this study.

Up to 242 patients will be included in this study over a period of approximately 2 years.

Patients must give written informed consent for study entry and the homocysteine blood test.

5.1 Inclusion criteria

Patients must fulfil ALL the inclusion criteria.

1. Written informed consent
2. Aged ≥ 18 years
3. Histologically or cytologically confirmed non-squamous NSCLC or MPM
4. Stage IIIB/IV disease.
5. Patients must be suitable to receive pemetrexed-based treatment with Cisplatin 50-75 mg/m^2^ (or Carboplatin AUC 5) or single agent pemetrexed therapy.
6. Patients must be able to take acid folic and vitamin B12 supplementation as normally required in clinical practice.
7. Estimated life expectancy of at least 12 weeks.

5.2 Exclusion criteria

Patients should not be enrolled in the study if they meet ANY of the exclusion criteria:

1. Pregnant or lactating women
2. Active infection
3. Inability or unwillingness to take vitamin supplementation
4. Serious systemic disorders incompatible with the study at the discretion of the investigator.
5. Patients receiving concomitant radical chemotherapy and radiotherapy (palliative or antalgic local radiotherapy is permitted)
6. Patients may have received previous lines of chemotherapy or targeted therapy as long as no vitamin supplementation was given.

**6. Study Treatment and Procedures**

6.1 Treatment Administration

Oral Folic acid 400 mcg p.o. daily will start optimally at least 1 week prior to the 1^st^ cycle of treatment but can start on day of first treatment.

Vitamin B12 1000 mcg i.m. will be administered as an intramuscular injection before chemotherapy and every 9 weeks.

Compliance with vitamins will be assessed at each treatment cycle.

Vitamin supplementation will continue until death, worsening of clinical condition, inability to take in oral tablets or patient consent withdrawal.

6.2 Concomitant Medications

Patients are allowed to receive full supportive care therapies concomitantly during the study. Palliative radiation therapy is permitted for irradiating small areas of painful metastases that cannot be managed adequately using systemic or local analgesics.

6.3 Chemotherapy Treatment

Patients must be suitable for chemotherapy treatment with standard non trial regimens e.g Pemetrexed 500 mg/m^2^ day 1 with either Cisplatin 50-75 mg/m^2^ or Carboplatin AUC 5 or single agent Pemetrexed every 21 days. See Lung unit guidelines for regimen details.

In case of toxicities G3 or more, these should be managed according to local guidelines

6.4 Treatment of Depression

Those identified as having depression with a Hospital and Anxiety Depression score of >7 will have their medical notes reviewed by the consultant psychiatrist at the Royal Marsden Hospital and management strategies eg antidepressant medication suggested. Follow up of their depressive condition will be by the lung unit team or via referral to the patient’s GP.

**7. Study Organisation**

The investigator is responsible for ensuring the study is conducted in accordance with the procedures and evaluations described in this protocol.

In accordance with the principles of GCP, the Investigator will maintain complete, accurate, legible and easily retrievable data. Such data shall also be secured in order to prevent loss of data.

CRFs for the recording and collecting of data will be developed along the lines of other trials run at RMH/ICR. All forms will be completed by the site staff in a legible manner using black ink. The investigator or designee must complete CRFs within a reasonable time period after data collection.

Corrections to data on CRFs will only be made by crossing out the incorrect data with a single line and writing the correct data next to the deleted data. The incorrect data must never be obliterated using correction fluid or similar preparations. Each correction will be initialed and dated by the person making the correction. The investigator will sign and date all CRFs to indicate that, to his/her knowledge, the data contained in the CRF are complete and accurate.

The investigator will maintain adequate and accurate records to enable the conduct of the study to be fully documented and the study data to be subsequently verified. These documents include Investigator’s Study Files and patient clinical source documents.

The investigator will ensure the Study Files are maintained with the CRFs and protocol/amendments, LREC and regulatory approvals with associated correspondence, informed consents, study drug records, staff curriculum vitae and authorization forms, all correspondence and other appropriate documents.

Patient clinical source documents may include, but are not limited to, patient hospital/clinic records, physician’s and nurse’s notes, appointment book, original laboratory reports, radiograph, pathology and special assessment reports and consultant letters.

Monitoring will be according to local guidelines in development.

The investigator is responsible for ensuring adequate accountability of all used and unused study drug. All drug supplies and associated documentation will be reviewed and verified according to local guidelines. The study site is responsible for the disposal and/or destruction of all unused study drug supplies, according to the site’s standard operating procedures.

All clinical study documents will be retained by the chief investigator for at least 5 years after the conclusion of the trial and in accordance with local procedures, Medicines for Human Use (Clinical Trials) Regulations (2004) and Amended Regulations (2006).

Principles of GCP require independent inspection of clinical program activities. Such inspections may be performed at any time, before, during and/or after the study. The investigator and study staff are responsible for maintaining a comprehensive and accurate filing system of all study-related documentation that will be suitable for inspection at any time by regulatory agencies.

The investigator will ensure that this study is conducted in full compliance with the current version of the “Declaration of Helsinki”, principles of GCP, and with the laws and regulations of the country in which the research is conducted, whichever affords the greater protection to the study patient.

The investigator will submit the site-specific informed consent form for this study to LREC. The investigator is responsible for obtaining written, informed consent(s) from each patient interested in participating in this study prior to conducting any study-related procedures. Written informed consent should be obtained after adequate, thorough and clear explanation of the aims, methods, objectives, potential hazards of the study as well as any use of the patient’s genetic information from the study. The investigator will use the most current LREC approved consent form for documenting written informed consent. Each informed consent will be appropriately signed and dated by the patient and the person obtaining consent. The investigational site must retain the original signed consent and provide a copy to the patient.

The RMH/ICR, the investigator, and/or the regulatory authorities reserve the right to terminate the study at any time. Should this be necessary, all parties will formulate and coordinate termination procedures. In terminating the study, LREC and the Investigator will assure that patients’ safety and rights are carefully protected.

RMH/ICR will determine the appropriate local, national, and/or regional regulatory approval(s) that need to be obtained in order to conduct this study.

Information concerning the protocol, the study information, patient medical information, patent applications, and processes, scientific data or other pertinent information as a result of this study is confidential and remains the property of RMH.

The investigator and the study center will adhere to all applicable laws relating to the protection of patient information. All personnel will handle patient data in a confidential manner in accordance with applicable regulations governing clinical research. Information generated as a result of a patient’s participation in this study may be disclosed to third parties for research and regulatory purposes in any country as determined by RMH. However, patients will not be individually identified but will be referred to by the assigned number and the patient’s initials.

After conclusion of the study, investigators in this study may make oral presentations of study results or publish such results in scientific journals or other scholarly media and an abstract, manuscript or presentation prepared.

As stated in the Patient Information Sheet, there are no special compensation arrangements if patients are harmed by taking part in the study. If harm is due to someone’s negligence, then patients may have grounds for legal action against the Royal Marsden NHS Trust at their own expense. The normal National Health Service complaints procedure mechanism is available to all patients for all matters.

**8. Study Procedures**

Start date is considered the date of the first patients enrolled and it expected to be around 1.9.2013. Dropouts (i.e. those with either no homocysteine level or those with blood homocysteine level but no toxicity data during 6 weeks) will be replaced until 242 patients with a homocysteine level are evaluable. The end of the study will be denoted as the date of the last clinic visit of the last patient participating in the clinical trial. All patients will be followed for survival.

**9. Study Assessment**

9.1 Baseline/Screening assessment

- FBC/biochemistry

- PS

- presence of symptoms

- presence of depression

- concomitant medications

9.2 Homocysteine assessment

Homocysteine plasma level will be measured before the second cycle of chemotherapy , after at least 3 weeks (21 days) of vitamin supplementation.

9.3 Toxicity assessment

Patients undergoing a treatment delay/ dose reduction/ drug change or hospitalization will be recorded before the second and third cycle. Toxicities will be reported using NCIC Common Toxicity Criteria (CTCAE v 4.0) at the same time points. All toxicities will be recorded. Preexisting symptoms will not be considered toxicities unless they deteriorate and this is considered due to the treatment e.g baseline grade 1 nausea which deteriorates to a grade 3 nausea at 6 weeks will be regarded as a grade 3 toxicity. However Grade 1 tumour related pain which deteriorates to grade 3 pain will not be considered a toxicity. If there is any conflict in allocating a symptom as disease or treatment related, the CI (MOB) will make a decision on this. The degree of toxicity will be taken as the total number of any individual grade 3-4 episodes recorded during both cycles (death and stopping treatment for any reason both counting as one episode). A grade 3 toxicity progressing to grade 4 during the same episode will be considered a grade 4.

9.4 Depression assessment

The Hospital Anxiety and Depression Score (HAD score) will be administered before the second cycle of chemotherapy , after at least 3 weeks (21 days) of vitamin supplementation.

9.5 Post-treatment assessment

Overall survival

Follow-up period: 1 year post entry of last patient.

**10. Evaluation of Outcome**

10.1 Primary endpoint:

Proportion of patients undergoing a treatment delay/ dose reduction/ drug change or hospitalisation during the first 6 weeks of chemotherapy. Treatment delay for administration reasons will be ignored.

These will be assessed on day 1 cycle 2 and day 1 cycle 3 i.e. over a 6 week period. They will be summated at one time point on day 1 cycle 3 after 6 weeks.

10.2 Secondary endpoints:

1. Proportion of patients with any grade 3-4 toxicity (except alopecia) or death within the first 2 cycles of chemotherapy i.e. assessed on day 1 cycle 3 after six weeks.

Toxicities will be assessed on day 1 cycle 2 and day 1 cycle 3 i.e. over a 6 week period. They will be summated at one time point on day 1 cycle 3 after 6 weeks.

2. Total number of grade 3-4 toxicities experienced by patients during the first 6 weeks of chemotherapy in those with homocysteine level <10 micromol/l and those with homocysteine level ≥10 micromol/l.

3.To compare OS in those with homocysteine level <10 micromol/l and those with homocysteine level 10 micromol/l or greater.

4. Toxicity and overall survival according to performance status 0,1 versus 2

5. Overall survival of NSCLC compared to mesothelioma

6. Overall survival of whole group consented to include all patients even those with/without homocysteine level but no or less than 6 weeks toxicity data. This includes all patients registered onto the study including dropouts, who may have died before the blood test at 3 weeks or died before toxicity assessment at 6 weeks.

7. To compare depression levels (rated as HAD-D >7) in those with homocysteine level <10 micromol/l and those with homocysteine level ≥ 10micromol/l.

8. To compare total length of stay in hospital (number of days summated for all hospital stays over the six week period) in those with homocysteine level < 10 micromol/l and those with homocysteine levels >/= 10 micromol/l.

**11. Adverse Events (gSOP-05-04, 5^th^ December 2011, RMH/ICR)**

This section describes the reporting of serious adverse events/reactions and the expected toxicities of the chemotherapy agents used in this protocol. The section should be read in conjunction with the Royal Marsden generic SOP-05-04 (5^TH^ December 2011) reporting for RMH/ICR sponsored CTIMP trials.

#### 11.1 Adverse event

An Adverse Event (AE) is defined as ‘any untoward medical occurrence in a subject to whom a medicinal product has been administered, including occurrences which are not necessarily caused by or related to that product’. An Adverse Drug Reaction (ADR) is ‘any untoward and unintended response in a subject to an investigational medicinal product which is related to any dose administered to that subject’.

This is a non-interventional trial and the chemotherapy is prescribed in the usual manner and in line with current practice. Therefore adverse reactions associated with the chemotherapy will be recorded on the CRF but do not require reporting as serious adverse events.

11.2 Serious adverse event

A Serious Adverse Event (SAE) or a Serious Adverse Reaction (SAR) is any event that results in one of more of the following:

- Death. Death is only reportable as an SAE if occurring during the study period (the first two cycle of chemotherapy and is not thought to be due to progressive cancer).
- Life-threatening situation
- Inpatient hospitalization or prolongation of existing hospitalization – for reasons other than lung cancer.
- Persistent or significant disability/incapacity - – due to any cause other than lung cancer
- Congenital anomaly or birth defect
- An important medical event not related to lung cancer disease progression.

Although this is a non-interventional trial and the chemotherapy is prescribed in the usual manner and in line with current practice, toxicity from chemotherapy will be recorded on the CRF and SAEs reported as they are part of the study endpoints.

11.3 Suspected Unexpected Serious Adverse Reactions (SUSARs)

An unexpected adverse drug reaction is an adverse reaction; the nature or severity of which is not consistent with the applicable product information (e.g. Investigator’s Brochure). However, an event which is listed in the product information but which occurs in a more specific or severe form than documented would be considered unexpected.

11.4 What should be reported?

All Serious Adverse Events shall be reported to the Chief Investigator within 24 hours in accordance with the Royal Marsden Hospital Standard Operating Procedures (SOP). All serious adverse events should be recorded and evaluated by the Chief Investigator for seriousness, causality, severity and expectedness. All unexpected SAEs and SUSARs must be reported to the Royal Marsden R&D office in accordance with the Royal Marsden Hospital SOP, using the Notification of Serious Adverse Events/SUSARs form available on the Royal Marsden intranet.

**Serious adverse events should be reported from the point at which cycle 1 has been administered on day 1 of the first cycle of chemotherapy up to 21 days after the second cycle of chemotherapy (i.e. the study period).**

**12. Statistical Considerations**

12.1 Sample size estimation

This will be an observational single arm study with 242 patients included.

All patients will have advanced stage lung cancer or mesothelioma planned to receive standard chemotherapy with vitamin B12 and folate supplementation.

A first and only homocysteine sample will be taken on day 1 cycle 2 of chemotherapy.

The primary endpoint will be the proportion of patients undergoing a treatment delay/ dose reduction/ drug change or hospitalisation within the first 2 cycles of chemotherapy i.e. assessed on day 1 cycle 3 after 6 weeks. Wepredict that those with UNSS (unsatisfactory supplementation i.e. Homocysteine level ≥10 micromol/l) will show a treatment delay/ dose reduction/ drug change or hospitalisation rate of 40 % and SS (successful supplemented i.e. homocysteine <10 micromol/l) will be at 15%. In order to detect a difference in treatment delay/ dose reduction/ hospitalisation rate between the two groups of 40% in UNSS compared to 15% in SS, with 80% power and 2-sided alpha of 5%, a total of 98 patients will be required i.e. 49 per group.

For the secondary endpoint of the proportion of patients with any grade 3-4 toxicity (except alopecia) or death within the first 2 cycles of chemotherapy i.e. assessed on day 1 cycle 3 after six weeks, we predict about 50% of patients will fall into the category of UNSS and 50% SS on day 1 cycle 2. We would predict the UNSS will show a toxicity rate of 30% and SS will be at 15%. The percentage will be the proportion of patients with any grade 3-4 toxicity on any occasion during the 6 weeks of observation.

In order to detect a difference in toxicities between the two groups of 30% in UNSS compared to 15% in SS, with 80% power and 2-sided alpha of 5%, a total of 242 patients will be required i.e. 121 per group.

This number of patients would also give us the power to detect a survival difference between the groups of homocysteine level <10 micromol/l and those with homocysteine level 10 micromol/l or greater which is a secondary endpoint.

The results from the previous study showed expected median survival of 15.7 months in SS group compared to 9.2 months in UNSS. With 115 patients in each group (total of 230 patients in study), we can detect a 1-year survival difference of 59% in SS compared to 41% in UNSS with hazard ratio = 0.59 and 110 events, assuming no dropouts (based on α_2 – sided_ = 5%, power = 80%).

Dropouts would be replaced until 242 patients with a homocysteine level are evaluable. Patients are still evaluable if they do not receive cycle 2 of chemotherapy as long as they have had a homocysteine level measured and the toxicity from the 1^st^ cycle is available.

12.2 Analysis of primary endpoint

The number of patients undergoing a treatment delay/ dose reduction/ drug change or hospitalisation during the first 6 weeks of chemotherapy between the two groups of homocysteine level <10 micromol/l and those with homocysteine level ≥10 micromol/l will be reported as a proportion with 95% confidence interval. Differences in patient numbers for treatment delay/ dose reduction etc. between the two homocysteine groups will be compared using a Chi-square or Fisher’s exact test. This will be assessed as (patients having a treatment delay/ dose reduction/ drug change or hospitalisation) vs. (patients not having treatment delay/ no dose reduction/ no drug change or no hospitalisation) between the two groups.

12.3 Analysis of secondary endpoints

The maximum grade of toxicity experienced by each patient during the first six weeks of chemotherapy will be calculated. Number of patients with grade 3-4 toxicity (except alopecia) or death between the two groups of homocysteine level <10 micromol/l and those with homocysteine level ≥10 micromol/l will be reported as a proportion with 95% confidence interval. Differences in grade 3-4 toxicities between the two homocysteine groups will be compared using a Chi-square or Fisher’s exact test. Toxicities will be assessed as grade <3 vs. grade ≥3.

Toxicities will be tabulated by type and grade of toxicity for each patient. Total number of grade 3-4 toxicities experienced by each patient during the first 6 weeks of chemotherapy will be reported between the two groups of homocysteine level <10 micromol/l and those with homocysteine level ≥10 micromol/l. Histogram plots will be presented for both groups.

Overall survival (OS) will be measured from the start of chemotherapy treatment until death from any cause or else censored at last known follow-up date. OS will be illustrated by means of Kaplan Meier curves and the logrank statistic will be used to compare survival differences between the groups listed. Median survival will be presented with 95% confidence intervals.

Differences in toxicities between performance status 0,1 versus 2 will be compared using a Chi-square or Fisher’s exact test.

Number of patients with a HAD-D score > 7 between the two groups of homocysteine level <10 micromol/l and those with homocysteine level ≥10 micromol/l will be reported as a proportion with 95% confidence interval. Any differences in scores between the two homocysteine groups will be compared using a Chi-square or Fisher’s exact test. HAD-D scores will be assessed as score ≤7 vs. score >7.

Total length of stay in hospital (number of days summated for all hospital stays over the six week period) in those with homocysteine level < 10 micromol/l and those with homocysteine levels >/= 10 micromol/l will be compared descriptively for the two groups.

A two-sided 5% level of significance will be used.

12.4 Timing of analysis

After the first 98 patients have finished 6 weeks of treatment, the primary analysis will be reported and a p-value calculated for differences between the groups. If this is significant then no other endpoints to be reported at this stage and recruitment will continue to 242 patients. If it is non-significant then recruitment stops and all remaining toxicity/ HADS secondary endpoints to be analysed after 98 patients.

Assuming the primary endpoint was found to be significant, the remaining toxicity/HADs secondary endpoints will be analysed after all 242 evaluable patients have reached 6 week assessment point. The primary endpoint will be reported again in this group of 242 patients but the test for difference will not be repeated.

The secondary endpoints for survival will be reported only after all patients have had 1 year of follow-up.

**13. References**

[1] Shih C, Habeck Ll, Mendelsohn LG et al. Multiple folate enzyme inhibitor: mechanisms pf a novel pyrrolopyrimidine-based antifolate LY231514 (MTA). Adv. Enzyme Regul. 38:135-52, 1998.

[2] Hanauske A, Chen V, Paoletti P et al. Pemetrexed disodium: a novel antifolate clinically active against multiple solid tumors. Oncologist 6:363-73, 2001.

3. Scagliotti GV, Parikh P, von Pawel J et al Phase III study Comparing Cisplatin Plus Gemcitabine With Cisplatin Plus Pemetrexed in Chemotherapy-Naïve Patients With Advanced-Stage Non-Small-Cell Lung Cancer. J Clin Oncol. 26 (21): 3543 – 3551. 2008

4. Zukin M, Barrios HC, Pereira JR et al. Randomized Phase III Trial of Single-Agent Pemetrexed Versus Carboplatin and Pemetrexed in Patients With Advanced Non–Small-Cell Lung Cancer and Eastern Cooperative Oncology Group Performance Status of 2. J Clin Oncol. 31 (23): 2849-2853. 2012

[5] Branda RF, Nigels E, Lafayette AR et al. Nutritional folate status influences the efficacy and toxicity of chemotherapy in rats. Blood 92:2471-76, 1998.

[6] Grindey GB, Alati T, Shih C. Reversal of the toxicity but not the antitumor activity of lometrexol by folic acid. Proc. Am. Assoc. Cancer Res. 32:324, 1991.

[7] Alati T, Shih C, Pohland RC et al. Evaluation of the mechanism(s) of inhibition of the toxicity, but not the antitumor activity of lometrexol (DDATHF) by folic acid. Proc. Am. Assoc. Cancer Res. 33:407, 1992.

[8] Wedge SR, Laohavinij S, Taylor GA, et al. Clinical pharmacokinetics of the antipurine antifolate (6R)-5, 10-dideaza-5,6,7,8-tetrahydrofolic acid (lometrexol) administration with an oral folic acid supplement. Clin Cancer Res. 1:1479-86, 1995.

[9] Lucock MD, Daskalakis I, Schorah C et al. Folate-homocysteine interrelations: potential new markers of folate status. Mol. Genet. Metab. 67:23-35, 1999.

[10] Stabler SP, Marcell PD, Savage DG et al. Elevation of total homocusteine in the serum of patients with cobalamin or folate deficiency detected by capillary gas chromatography-mass spectrometry. J. Clin. Investig. 81:466-74, 1988.

[11] Niyikiza C, Baker SD, Seitz DE, et al. Homocysteine and methylmalonic acid: markers to predict and avoid toxicity from pemetrexed therapy. Mol. Cancer Ther. 1:545-52, 2002.

[12] Homocysteine Lowering Trialists’ Collaboration, Lowering blood homocysteine with folic acid based supplements: meta-analysis of randomized trials. BMJ 316:894-98, 1998.

[13] Vogelzang NJ, Rusthoven JJ, Symanowsky J et al. Phase III study of pemetrexed in combination with cisplatin versus cisplatin alone in patients with malignant pleural mesothelioma. J. Clin. Oncol. 14:2636-44, 2003.

14. Temel JS, Greer JA, Muzikansky A et al. Early Palliative Care for Patients with Metastatic Non–Small-Cell Lung Cancer. New Eng J Med. 363: 733-742, 2010

15. Walker J, Hanse CH, Martin P et al. Prevalence, associations, and adequacy of treatment of major depression in patients with cancer: a cross-sectional analysis of routinely collected clinical data. Lancet Psych. 2014

[epub ahead of print] doi: 10.1016/S2215-0366(14)70313-X.

16. Lyons L, ElBeltagy M, Umka J, Markwick R, Startin C, et al. Fluoxetine reverses the memory impairment and reduction in proliferation and survival of hippocampal cells caused by methotrexate chemotherapy. Psychopharmacology. 215: 105–115. 2011

17. Zigmond AS, Snaith RP. The Hospital Anxiety and Depression Scale. Acta Psychiatr Scand. 67:361 – 70. 1983

18. Bjelland I, Dahl AA, Tangen Haug T et al. The validity of the Hospital Anxiety and Depression Scale An updated literature review. J Psychosomatic Res. 52: 69–77. 2002

**14. Appendices**

Appendix 1: Chemotherapy regimens – including single agents iv and oral. See lung unit guidelines for more details.

PEMETREXED-BASED REGIMENS (every 21 days)

| Cisplatin 75 mg/m^2^ | i.v. | In 2 hours | Day 1 |
| --- | --- | --- | --- |
| Pemetrexed 500 mg/m^2^ | i.v. | In 10 minutes | Day 1 |

| Carboplatin AUC5 | i.v. | In 1 hour | Day 1 |
| --- | --- | --- | --- |
| Pemetrexed 500 mg/m^2^ | i.v. | In 10 minutes | Day 1 |

| Single Agent Pemetrexed 500 mg/m^2^ | i.v. | In 10 minutes | Day 1 |
| --- | --- | --- | --- |

Appendix 2: Adverse events of chemotherapy regimens

**CISPLATIN**

The incidences of adverse events relating to cisplatin are based on information regarding cisplatin from the Bristol Myers Squibb clinical trials database.

| SUMMARY OF ADVERSE EVENTS | |
| --- | --- |
| **ONSET** | **SIDE EFFECT**  may be life-threatening; side effects in **bold** type are common |
| **IMMEDIATE** (hours to days) | anaphylaxis (1-20%) |
|  | **nausea and vomiting**  (most patients, moderate to severe, onset 1-4 hours, duration 1-7 days) |
| **EARLY** (days to weeks) | low WBC, RBC, platelets (25-30%, myelosuppression, nadir 18-23 days, recovery 39 days) |
|  | **kidney problems** (28-36%, toxic nephropathy, hypomagnesemia, electrolyte disturbances) |
|  | nausea and vomiting |
|  | heart problems (electrocardiographic changes, rare) |
|  | liver problems (elevated liver function tests, rare) |
|  | blood problems (hemolytic anemia) |
|  | CNS problems (acute encephalopathy, rare) |
| **DELAYED/LATE** (weeks to years) | **nerve problems** (peripheral neuropathy) |
|  | CNS problems (acute encephalopathy, rare) |
|  | eye problems (retinopathy, optic neuropathy) |
|  | hearing problems (24%, ototoxicity) |
|  | infertility |
|  | Raynaud's syndrome (rare) |

**(Table above from Cancer Drug Manual© 1994)**

**Nephrotoxicity:** Dose-related and cumulative renal insufficiency is the major dose-limiting toxicity of cisplatin. Renal toxicity has been noted in 28% to 36% of patients treated with a single dose of 50mg/m^2^. It is first noted during the second week after a dose and is manifest by elevations in blood urea nitrogen and Creatinine, serum uric acid and/or a decrease in Creatinine clearance.

**Ototoxicity**: Ototoxicity has been observed in up to 31% of patients treated with a single dose of cisplatin 50mg/m^2^ and is manifested by tinnitus and/or hearing loss in the high frequency range (4000 to 8000 Hz). Decreased ability to hear normal conversational tones may occur occasionally. Deafness after the initial dose has been reported rarely. Hearing loss can be unilateral or bilateral and tends to become more frequent and severe with repeated doses. It is unclear whether cisplatin induced ototoxicity is reversible.

**Haematological**: Myelosuppression is seen in 25% to 30% of patients, with nadirs in circulating platelets occurring between days 18-23 and most patients recovering by day 39. Leukopenia and thrombocytopenia are more pronounced at higher doses; anaemia occurs at approximately the same frequency and with the same timing. Fever and infection have also been reported in patients with neutropenia.

In addition to anaemia secondary to myelosuppression, a Coombs’ positive haemolytic anaemia has been reported.

The development of acute leukaemia coincident with cisplatin has rarely been reported in humans. In these reports cisplatin was generally given with other leukemogenic agents

**Gastrointestinal:** Marked nausea and vomiting occur in almost all patients treated with cisplatin and are occasionally so severe that the drug must be discontinued. Nausea and vomiting usually begin within 1 to 4 hours after treatment and lasts up to 24 hours. Various degrees of vomiting, nausea and/or anorexia may persist for up to 1 week after treatment. Delayed nausea and vomiting (begins or persists 24 hours or more after chemotherapy has occurred in patients attaining complete emetic control on the day of cisplatin therapy. Diarrhoea has been reported.

OTHER TOXICITIES

**Serum Electrolyte Disturbances:** Hypomagnesaemia, hypocalcaemia, hyponatraemia, hypokalaemia and hypophosphataemia have been reported to occur and are probably related to renal tubular damage. Tetany has occasionally been reported in those patients with hypocalcaemia and Hypomagnesaemia. Generally normal serum electrolyte levels are restored by administering supplemental electrolytes and discontinuing cisplatin. In appropriate anti-diuretic hormone syndrome has also been reported.

**Hypersensitivity:** Occasionally reported in patients exposed to cisplatin. Contraindicated in patients with a history of allergic reactions to cisplatin or other platinum-containing compounds. Anaphylactic-like reactions occurring within minutes of administration seen with prior exposure to cisplatin, and have been relieved with use of epinephrine, corticosteroids, and antihistamines.

**Hyperuricaemia:** Has been reported to occur at approximately same frequency as the increases in blood urea nitrogen and serum creatinine. Hyperuricaemia is more pronounced after doses > 50mg/m², with peak levels occurring generally between 3-5 days after dose. Allopurinol therapy effectively reduces uric acid levels.

**Neurotoxicity:** Neurotoxicities usually characterised by peripheral neuropathies has been reported. The neuropathies usually occur after prolonged therapy (4 to 7 months), however neurological symptoms have been reported to occur after a single dose. Although symptoms and signs of cisplatin neuropathy usually develop during treatment, symptoms may begin 3 to 8 weeks after the last dose of cisplatin, although this is rare. The neuropathy may progress further even after stopping treatment. Preliminary evidence suggests peripheral neuropathy may be irreversible in some patients.

Lhermitte’s sign, dorsal column myelopathy and autonomic neuropathy have also been reported. Loss of taste and seizures has also been reported.

Muscle cramps defined as localised, painful, involuntary skeletal muscle contractions of sudden onset and short duration have been reported and were usually associated in patients receiving relatively high cumulative dose of cisplatin and with a relatively advanced symptomatic stage of peripheral neuropathy.

**Ocular toxicity:** Optic neuritis, papilloedema and cerebral blindness have been reported infrequently in patients receiving standard recommended doses of cisplatin. Improvement and/or total recovery usually occurs after discontinuing cisplatin.

Blurred vision and altered colour perception manifests as a loss of colour discrimination, particularly in the blue-yellow axis. The only finding on funduscopic exam is irregular retinal pigmentation of the macular area.

**Hepatotoxicity** Transient elevations of liver enzymes, especially SGOT, as well as bilirubin have been reported to be associated with cisplatin administration at the recommended doses.

**Other Events** Other toxicities reported to occur infrequently are cardiac abnormalities, hiccups, elevated serum amylase and rash. Alopecia, malaise, and asthenia have been reported.

Local soft tissue toxicity has rarely been reported following extravasation of cisplatin. Severity of the local tissue toxicity appears to be related to the concentration of the cisplatin solution. Infusion of solutions with a concentration >0.5mg/mL may result in tissue cellulites, fibrosis and necrosis.

There are serious side effects associated with cisplatin, notably renal toxicity, emesis, neurotoxicity, bone marrow suppression and hearing loss. Damage to the kidneys can be minimized through the administration of continuous IV hydration along with diuretic drugs before and following the infusion of cisplatin. Similarly, several effective anti-emetic drugs protect the patient from the worst of nausea and vomiting. Testing of patient renal function, blood and hearing is recommended before each cycle of therapy

**CARBOPLATIN**

The incidences of adverse events relating to carboplatin are based on from SmPC

Myelosuppression is the dose limiting toxic reaction of carboplatin. It is generally reversible and not cumulative when carboplatin is used as a single agent at recommended frequencies of administration. Adverse reactions which have occurred in studies to date can be grouped under the following systems:

***Blood and the lymphatic system disorders***: Leucopenia (55%), thrombocytopenia (32%) and anaemia (59%) of patients. Transfusion support has been required in about 20% of patients. Haemolytic uraemic syndrome has been reported. Infectious complications and haemorrhagic complications have also been reported.

***Respiratory, thoracic and mediastinal disorders***: Pulmonary fibrosis has been reported very rarely, manifested by tightness of the chest and dyspnoea. This should be considered if a pulmonary hypersensitivity state is excluded (see General disorders below).

***Gastrointestinal disorders:*** Nausea and vomiting (53%), nausea only in 25%. Nausea and vomiting are generally delayed until 6 to 12 hours after administration of carboplatin, are readily controlled or prevented with antiemetics and disappear within 24 hours. Diarrhoea occurred in 6% and constipation in 3% of patients. Abdominal pain and cramps have also been reported.

***Nervous system disorders:*** Mild peripheral neuropathy occurred in 6% of patients and dysgeusia in less than 1% of patients. Parasthesias present prior to treatment, especially if caused by cisplatin, may persist or worsen during carboplatin therapy. (See Precautions).

***Eye disorders:*** Transient visual disturbances, sometimes including transient sight loss, have been reported rarely with platinum therapy. This is usually associated with high dose therapy in renally impaired patients.

***Ear and labyrinth disorders:*** A subclinical decrease in hearing acuity in the high frequency range (4000-8000 Hz), determined by audiogram, occurred in 15% of patients. Clinical ototoxicity also manifested itself as tinnitus (1% of patients). Hearing loss as a result of cisplatin therapy may give rise to persistent or worsening symptoms. At higher than recommended doses, in common with other ototoxic agents, clinically significant hearing loss has been reported to occur in paediatric patients when carboplatin is administered.

***Hepato-biliary disorders****:* Transient increases in liver enzymes have been reported in some patients. Alkaline phosphatase was increased in 30% of patients, with aspartate aminotransferase (15% patients) and elevated serum bilirubin (4% patients) occurring less frequently.

***Renal and urinary disorders:*** Renal toxicity is not usually dose limiting. However, a decrease in creatinine clearance is observed in approximately 25% of patients. A rise in uric acid (25%) and, less frequently, a rise in serum creatinine (7%) and blood urea nitrogen (16%) have also been observed. Impairment of renal function is more likely in patients who have previously experienced nephrotoxicity as a result of cisplatin therapy.

***General disorders****:* Rarely anaphylaxis and anaphylactic-like reactions have been reported including tachycardia, bronchospasm, dyspnoea, hypotension, wheezing, urticaria, facial oedema and facial flushing. Erythematous rash, fever and pruritis have been observed in less than 2% of patients treated. These were reactions similar to those seen after cisplatin therapy but in a few cases no cross-reactivity was present.

Decreased serum levels of magnesium (37% patients), potassium (16% patients) and calcium (5% patients) have occurred although not severe enough to cause clinical symptoms. Decreased serum sodium has also been reported although it is normally insufficient to require treatment. There have also been rare reports of hyponatraemia.

Asthenia is very commonly reported. Rare events have included alopecia (2%), a flu

like syndrome (1%) and reaction at the injection site (<1%). Cases of anorexia have been reported.

**PEMETREXED**

In clinical trials, the most common adverse reactions (incidence u20%) during therapy with pemetrexed as a single-agent were fatigue, nausea, and anorexia. Additional common adverse reactions (incidence u20%) during therapy with Pemetrexed when used in combination with cisplatin included vomiting, neutropenia, leukopenia, anemia, stomatitis/pharyngitis, thrombocytopenia, and constipation.

**Next table provides the frequency and severity of adverse reactions that have been reported in >5% of 839 patients with NSCLC who were randomized to study and received Pemetrexed plus cisplatin and 830 patients with NSCLC who were randomized to study and received gemcitabine plus cisplatin. All patients received study therapy as initial treatment for locally advanced or metastatic NSCLC and patients in both treatment groups were fully supplemented with folic acid and vitamin B12.**

| **Pemetrexed /cisplatin (N=839)** | | | | |  | |
| --- | --- | --- | --- | --- | --- | --- |
|  | **All Grades Toxicity (%)** | | **Grade 3-4 Toxicity (%)** | |  | |
| **All Adverse Reactions** | | 90 | | 37 | |  |
| **Laboratory** | | | |  | |  |
| **Hematologic** | | | |  | |
| Anemia | | 33 | | 6 | |  |
| Neutropenia | | 29 | | 15 | |  |
| Leukopenia | | 18 | | 5 | |  |
| Thrombocytopenia | | 10 | | 4 | |  |
| **Renal** | | | | | |  |
| Creatinine elevation | | 10 | | 1 | |  |
| **Clinical** | | | | | |  |
| **Constitutional Symptoms** | | | | | |
| Fatigue | | 43 | | 7 | |  |
| **Gastrointestinal** | | | | | |  |
| Nausea | | 56 | | 7 | |  |
| Vomiting | | 40 | | 6 | |  |
| Anorexia | | 27 | | 2 | |  |
| Constipation | | 21 | | 1 | |  |
| Stomatitis/Pharyngitis | | 14 | | 1 | |  |
| Diarrhea | | 12 | | 1 | |  |
| Dyspepsia/Heartburn | | 5 | | 0 | |  |
| **Neurology** | | | | | |  |
| Neuropathy-sensory | | 9 | | 0 | |  |
| Taste disturbance | | 8 | | 0c | |  |
| **Dermatology/Skin** | | | | | |  |
| Alopecia | | 12 | | 0c | |  |
| Rash/Desquamation | | 7 | | 0 | |  |

^a^ For the purpose of this table a cut off of 5% was used for inclusion of all events where the reporter considered a possible relationship to Pemetrexed.

^b^ Refer to NCI CTC Criteria version 2.0 for each Grade of toxicity.

^c^ According to NCI CTC Criteria version 2.0, this adverse event term should only be reported as Grade 1 or 2.

**Incidence 1% to 5%**

*Body as a Whole* — febrile neutropenia, infection, pyrexia

*General Disorders* — dehydration

*Metabolism and Nutrition* – increased AST/ALT

*Renal* – Creatinine clearance decrease, renal failure

*Special sense* – conjunctivitis

**Incidence Less than 1%**

*Cardiovascular* – arrhythmia

*General disorders* – chest pain

*Metabolism and nutrition* – increased GGT

*Neurology* – motor neuropathy

**Appendix 3:**

**Hospital Anxiety and Depression Scale (HADS)**

**Please choose one response from the four options given for each question.**

**Try to give an immediate response and not think for too long about each answer.**

**Please answer according to how you feel at this point in time.**

| A | **I feel tense or 'wound up':** |  |  |
| --- | --- | --- | --- |
|  | Most of the time |  | 3 |
|  | A lot of the time |  | 2 |
|  | From time to time, occasionally |  | 1 |
|  | Not at all |  | 0 |

| D | **I still enjoy the things I used to enjoy:** |  |  |
| --- | --- | --- | --- |
|  | Definitely as much |  | 0 |
|  | Not quite so much |  | 1 |
|  | Only a little |  | 2 |
|  | Hardly at all |  | 3 |

| A | **I get a sort of frightened feeling as if something awful is about to happen:** |  |  |
| --- | --- | --- | --- |
|  | Very definitely and quite badly |  | 3 |
|  | Yes, but not too badly |  | 2 |
|  | A little, but it doesn't worry me |  | 1 |
|  | Not at all |  | 0 |

| D | **I can laugh and see the funny side of things:** |  |  |
| --- | --- | --- | --- |
|  | As much as I always could |  | 0 |
|  | Not quite so much now |  | 1 |
|  | Definitely not so much now |  | 2 |
|  | Not at all |  | 3 |

| A | **Worrying thoughts go through my mind:** |  |  |
| --- | --- | --- | --- |
|  | A great deal of the time |  | 3 |
|  | A lot of the time |  | 2 |
|  | From time to time, but not too often |  | 1 |
|  | Only occasionally |  | 0 |

| D | **I feel cheerful:** |  |  |
| --- | --- | --- | --- |
|  | Not at all |  | 3 |
|  | Not often |  | 2 |
|  | Sometimes |  | 1 |
|  | Most of the time |  | 0 |

| A | **I can sit at ease and feel relaxed:** |  |  |
| --- | --- | --- | --- |
|  | Definitely |  | 0 |
|  | Usually |  | 1 |
|  | Not Often |  | 2 |
|  | Not at all |  | 3 |

| D | **I feel as if I am slowed down:** |  |  |
| --- | --- | --- | --- |
|  | Nearly all the time |  | 3 |
|  | Very often |  | 2 |
|  | Sometimes |  | 1 |
|  | Not at all |  | 0 |

| A | **I get a sort of frightened feeling like 'butterflies' in the stomach:** |  |  |
| --- | --- | --- | --- |
|  | Not at all |  | 0 |
|  | Occasionally |  | 1 |
|  | Quite Often |  | 2 |
|  | Very Often |  | 3 |

| D | **I have lost interest in my appearance:** |  |  |
| --- | --- | --- | --- |
|  | Definitely |  | 3 |
|  | I don't take as much care as I should |  | 2 |
|  | I may not take quite as much care |  | 1 |
|  | I take just as much care as ever |  | 0 |

| A | **I feel restless as I have to be on the move:** |  |  |
| --- | --- | --- | --- |
|  | Very much indeed |  | 3 |
|  | Quite a lot |  | 2 |
|  | Not very much |  | 1 |
|  | Not at all |  | 0 |

| D | **I look forward with enjoyment to things:** |  |  |
| --- | --- | --- | --- |
|  | As much as I ever did |  | 3 |
|  | Rather less than I used to |  | 2 |
|  | Definitely less than I used to |  | 1 |
|  | Hardly at all |  | 0 |

| A | **I get sudden feelings of panic:** |  |  |
| --- | --- | --- | --- |
|  | Very often indeed |  | 3 |
|  | Quite often |  | 2 |
|  | Not very often |  | 1 |
|  | Not at all |  | 0 |

| D | **I can enjoy a good book or radio or TV program:** |  |  |
| --- | --- | --- | --- |
|  | Often |  | 3 |
|  | Sometimes |  | 2 |
|  | Not often |  | 1 |
|  | Very seldom |  | 0 |

**Thank you**
